# Investigation on Stray-Capacitance Influences of Coaxial Cables in Capacitive Transducers for a Space Inertial Sensor

**DOI:** 10.3390/s20113233

**Published:** 2020-06-06

**Authors:** Jianbo Yu, Chengrui Wang, Ying Wang, Yanzheng Bai, Ming Hu, Ke Li, Zhuxi Li, Shaobo Qu, Shuchao Wu, Zebing Zhou

**Affiliations:** 1 MOE Key Laboratory of Fundamental Physical Quantities Measurement, Hubei Key Laboratory of Gravitation and Quantum Physics, PGMF, School of Physics, Huazhong University of Science and Technology, Wuhan 430074, China; yujb@hust.edu.cn (J.Y.); D201880109@hust.edu.cn (C.W.); ywang@alumni.hust.edu.cn (Y.W.); D201377062@hust.edu.cn (K.L.); lizhuxi@hust.edu.cn (Z.L.); qushaobo@hust.edu.cn (S.Q.); scwu@hust.edu.cn (S.W.); zhouzb@mail.hust.edu.cn (Z.Z.); 2 Institute of Geodesy and Geophysics, Chinese Academy of Sciences, Wuhan 430077, China; huming@whigg.ac.cn

**Keywords:** space inertial sensor, capacitive transducer, stray-capacitance, coaxial cables

## Abstract

Ultra-sensitive inertial sensors are one of the key components in satellite Earth’s gravity field recovery missions and space gravitational wave detection missions. Low-noise capacitive position transducers are crucial to these missions to achieve the scientific goal. However, in actual engineering applications, the sensor head and electronics unit usually place separately in the satellite platform where a connecting cable is needed. In this paper, we focus on the stray-capacitance influences of coaxial cables which are used to connect the mechanical core and the electronics. Specially, for the capacitive transducer with a differential transformer bridge structure usually used in high-precision space inertial sensors, a connecting method of a coaxial cable between the transformer’s secondary winding and front-end circuit’s preamplifier is proposed to transmit the AC modulated analog voltage signal. The measurement and noise models including the stray-capacitance of the coaxial cable under this configuration is analyzed. A prototype system is set up to investigate the influences of the cables experimentally. Three different types and lengths of coaxial cables are chosen in our experiments to compare their performances. The analysis shows that the stray-capacitance will alter the circuit’s resonant frequency which could be adjusted by additional tuning capacitance, then under the optimal resonant condition, the output voltage noises of the preamplifier are measured and the sensitivity coefficients are also calibrated. Meanwhile, the stray-capacitance of the cables is estimated. Finally, the experimental results show that the noise level of this circuit with the selected cables could all achieve 1–2 × 10^−7^ pF/Hz^1/2^ at 0.1 Hz.

## 1. Introduction

The high precision inertial sensor is one of the key payloads in satellite Earth’s gravity field recovery missions [1] and space gravitational experiments [2,3,4,5]. The capacitive position transducer is crucial to the success of these missions where it measures the relative motion between the test mass (TM) and the electrodes frame. The Office National d’Etudes et de Recherches Aérospatiales (ONERA) has developed it with a performance of 10^−7^ pF/Hz^1/2^ level above 0.01 Hz [6]. A terrific stable capacitive sensing circuit of the bandwidth down to 0.1 mHz has carefully been studied [7,8,9,10] for space gravitational wave detection mission LISA, and in-flight tests have achieved the anticipated goal at mHz bandwidth in LISA Pathfinder mission [11,12]. In our lab, a similar capacitive position transducer has also been developed, the effect of the excitation frequency in differential capacitive sensing, the prototype sensing circuit, and a pre-scanning and adjustment method are investigated, respectively [13,14,15,16].

In actual space engineering applications, the sensor head and the electronics are usually two separate equipment in the satellite. For example, concerning the capacitive position transducer, the mechanical core of the gradiometer in the GOCE mission consists of a low expansion carbon-carbon sandwich panels and six electrostatic accelerometers’ sensor heads [17,18]. However, the electronics of the gradiometer needs to operate far from the mechanical core in the satellite dimension. In space gravitational detection missions, the gravitational reference sensor system consists of a cubic test mass and surrounding electrodes assembled in a vacuum chamber. The front-end electronics unit needs to detect the signal outside the vacuum chamber. So coaxial cables are used to connect those two pieces of equipment to achieve the measurement and so on. The influences of the stray-capacitance of the cables need to be investigated. In some applications, the analysis of capacitive sensor noise [19,20,21,22,23] and the influences of stray-capacitance [24,25,26,27,28,29,30] have been considered in their experiments.

Due to the low-noise and high-stability requirement of the capacitive sensor for inertial sensors used in the above missions, the transducer structure based on the differential transformer bridge is usually used. Based on this type of sensor, the emphasis on this study aims at investigating the stray-capacitance influences of the coaxial cables on the capacitive position transducer where the cables connect the sensor head and the electronics. Different from the connecting method between the sensor head and the transformer bridge, a connecting method between the transformer’s secondary winding and the preamplifier of the front-end circuit is proposed, and its purpose is to transmit the signal that is modulated onto an AC frequency carrier. The signal and noise models of the front-end circuit under this configuration is analyzed. A prototype circuit board with three different types and lengths of coaxial cables is realized. The performance of the whole transducer under nine different conditions is measured, compared, and analyzed. It indicates that the type and length of coaxial cables are not so important relative to the resonant work condition under our connecting approach. All the noise level could achieve 1–2 × 10^−7^ pF/Hz^1/2^ at 0.1 Hz under the optimal resonant condition even with a two-meter-long connecting cable. It is useful for estimating the stray-capacitance of selected cables and tuning the circuits in actual engineering applications.

## 2. Connecting Method of Coaxial Cables and Its Stray-Capacitance Analysis

### 2.1. The Principle of the Capacitive Transducer Focusing on the Cable’s Connecting Method

**Figure 1:**
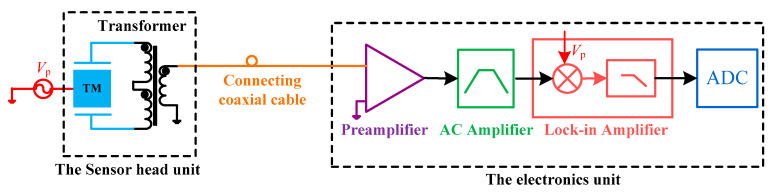
Schematic diagram of capacitive sensing circuit with a coaxial cable.

The schematic diagram of the capacitive sensing circuit based on the differential transformer bridge for space inertial sensors is shown in Figure 1. Considering the actual application for a satellite environment, the sensor head unit and the electronics unit are separate. In our design, a connecting coaxial cable is adopted here as a transmission channel to combine the transformer’s secondary winding and the preamplifier. Theoretically, it can also increase the connecting cable between the sensor head and the transformer bridge, namely between the electrodes and the two primary windings of the transformer [7,11]. The cables are used as the extended connecting wire of the electrodes, where the stability and symmetry influences of the cables need to be more concerned.

In our design, a long coaxial cable is used specifically to transmit the modulated differential capacitance signal including the position information between the TM and the electrodes. It also shows that a three-winding transformer needs to be configured near the sensor head to directly transmit the AC signal. This connecting method theoretically intends to better suppress the low-frequency influences of the cables compared with the method by extending the transmission wires of the electrodes directly, especially when the cable is much longer. So in this paper, the experiments focus on investigating the influences of the cables using the method in Figure 1.

Then the electronics unit includes a preamplifier, a band-pass amplifier, a lock-in amplifier (a demodulator, and a low-pass filter circuit), and an ADC device [6,14]. *V*_p_ is the pumping voltage signal with a frequency of 90 kHz in our design. Theoretically, the thermal noise of the front-end circuit (the transformer and preamplifier) of this sensor plays a dominant role, so only the measurement and noise model of the front-end circuit is introduced in detail.

**Figure 2:**
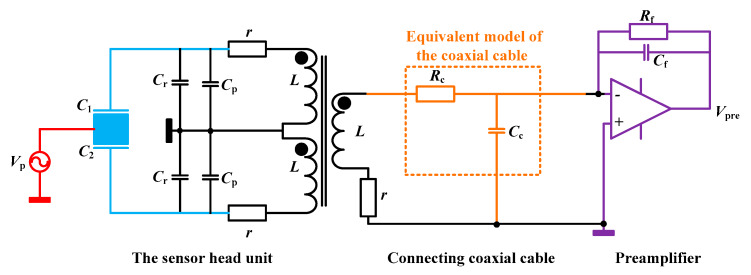
The signal model of the transformer and preamplifier with a coaxial cable.

The equivalent measurement model of the transformer and preamplifier is shown in Figure 2. *C*_1_ and *C*_2_ represent the sensing capacitance. *C*_r_ is the tuning capacitance. *C*_p_ is the parasitic capacitance of the printed circuit board (PCB). L is the inductance of the transformer’s each identical winding. *R* is the equivalent AC resistance of the winding. *C*_f_ and *R*_f_ are the feedback capacitance and resistance. *R*_c_ and *C*_c_ are the equivalent loss resistance and stray-capacitance of the coaxial cable. The equivalent inductance of the cable is neglected in this model due to its smaller value relative to the transformer.

In order to get the main relationship between the transformer and the preamplifier, we assume a hypothesis of an ideal transformer and preamplifier working at the resonant condition. The signal output voltage of the preamplifier is proportional to the input differential capacitance which can be expressed as

(1) $$V_{\mathrm{pre}}\approx -\Delta C\frac{V_{p}}{C_{f}},$$

where $\Delta C=C_{1}-C_{2}$ is the differential capacitance signal which presents the relative motion of the TM.

### 2.2. The Noise Analysis on the Influences of Stray-Capacitance of the Cables

**Figure 3:**
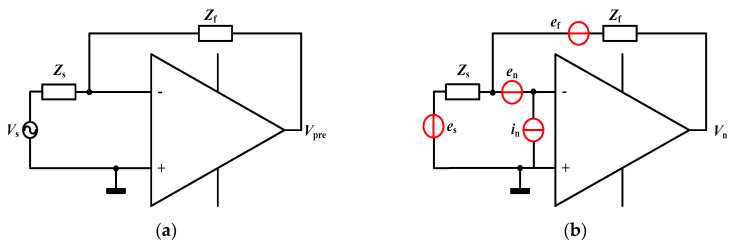
The equivalent function (**a**) and noise (**b**) model of the front-end circuit.

The total thermal noise needs to especially focus on the long cable because of its loss resistance and stray-capacitance. The differential transformer bridge with a long cable is equivalent to a voltage source vs. in series connection with the impedance *Z*_S_. The noise model is shown in Figure 3, where *Z*_f_ is the parallel connection of *C*_f_ and *R*_f_, *e*_s_ and *e*_f_ mean the thermal noise of the bridge and the feedback impedance, *e*_n_ and *i*_n_ represent the equivalent input voltage and current noise of the amplifier, respectively.

Neglecting the higher order small quantities, the equivalent impedance *Z*_S_ and the voltage vs. can be written as

(2) $$Z_{S}=\frac{LS}{1+(rC_{r}+rC_{c}+R_{c}C_{c})S+(2C_{r}+C_{c})LS^{2}},$$

(3) $$V_{S}=Z_{S}\Delta CV_{p}S,$$

where *S* is the Laplace variable. Considering above four equivalent noise sources, then the total output noise of the front-end circuit is given by

(4) $$V_{n}=\sqrt{e_{s}{}^{2}{\left|-\frac{Z_{f}}{Z_{s}}\right|}^{2}+e_{f}{}^{2}+e_{n}{}^{2}{\left|1+\frac{Z_{f}}{Z_{s}}\right|}^{2}+i_{n}{}^{2}{\left|Z_{f}\right|}^{2}}.$$

In order to insure the best performance, this circuit must work in a resonant condition. Theoretically, this constraint condition can be expressed as

(5) $$1+(2C_{r}+2C_{p}+C_{c})LS^{2}=0.$$

It is obvious that the equivalent stray-capacitance of the coaxial cable will directly act on the resonant frequency *ω*_p_. So here if we assume the stray-capacitance per unit length of the coaxial cable is *k*_c_ (F/m), and the distance is *l*_c_, then the total capacitance *C*_c_ = *k*_c_*l*_c_. Then the resonant condition equation can be written as

(6) $$1=(2C_{r}+2C_{p}+k_{c}l_{c})L\omega_{p}^{2}.$$

Considering the capacitance *C*_r_ in actual circuit is positive, we can get

(7) $$l_{c}\leq \frac{1}{k_{c}L\omega_{p}^{2}}.$$

It indicates that the length of the coaxial cable will be limited by the type of the cable, the transformer inductance, and the pumping voltage frequency.

Under the resonant condition, the theoretical analysis shows that the thermal noise of the bridge, namely the contribution of *e*_s_ dominates at the resonant frequency [14]. The equivalent capacitance detection thermal noise from the bridge and the transmission cable is given by

(8) $$C_{n,es}=\frac{1}{V_{p}}\sqrt{\frac{8k_{B}T}{\omega_{p}^{3}L}(\frac{1}{Q_{t}}+\frac{1}{Q_{c}})},$$

where *k*_B_ is Boltzmann constant, *T* presents the absolute temperature, $Q_{t}=\omega_{p}L/r$ is the quality factor of the transformer, and $Q_{c}=\omega_{p}L/R_{c}$ is equivalent quality factor due to the loss resistance.

So the influences of coaxial cables result in altering the circuit’s resonant condition and increasing the thermal noise. Besides, considering the real transformer and the amplifier which will slightly influence the tuning of resonance, the actual circuits must be tuned experimentally.

### 2.3. The Parameters and Potential Ability of the Prototype Circuit

In this paper, a prototype circuit board was realized with the designed parameters given in Table 1. The pumping frequency of the excitation signal is usually chosen from dozens of kHz to hundreds of kHz due to the frequency response of the transformer and the amplifier. In our experiment, this frequency is chosen to be 90 kHz.

**Figure 4:**
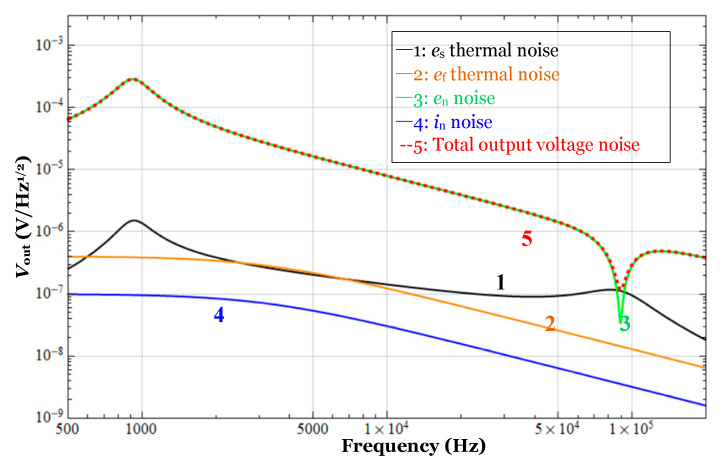
The noise curve estimation of the front-end circuit of the capacitive sensor with a coaxial cable.

If the resonant condition is met by adjusting the tuning capacitance to compensate for the stray-capacitance of the cables, then the contributions of each noise source are analyzed in Figure 4. The red curve is the total voltage of the front-end circuit, and the curves of black, brown, green, and blue represent the contributions of *e*_s_, *e*_f_, *e*_n_, *i*_n_, respectively. The expressions of *e*_s_ and *e*_f_ are 4kBTRe[ZS] and 4kBTRe[Zf], respectively, where the symbol Re means the real part of the impedance. So according to equation (4), the basic frequency response expression of each noise contribution is |ZfZs|4kBTRe[ZS], 4kBTRe[Zf], $\left|1+\frac{Z_{f}}{Z_{s}}\right|e_{n}$ and $\left|Z_{f}\right|i_{n}$, which mainly comes from *Z*_S_ and *Z*_f_. Meanwhile, the frequency response of the open-loop gain of the preamplifier is also considered and finally each curve is obtained by using mathematical software in Figure 4. Besides, the noise contribution from the loss resistance of the cable could be neglected due to the higher equivalent quality factor *Q*_c_.

Figure 4 shows that the total output voltage noise of the front-end circuit with a long coaxial cable is about 1.0 × 10^−7^ V/Hz^1/2^ at the pumping frequency where it is currently limited by the contributions of *e*_s_, and the equivalent capacitance detection noise level is about 1.2 × 10^−7^ pF/Hz^1/2^.

## 3. Experimental Investigation on Stray-Capacitance Estimation, Capacitive Transducer Calibration, and Noise Level Comparison

### 3.1. Experimental System Set up

**Figure 5:**
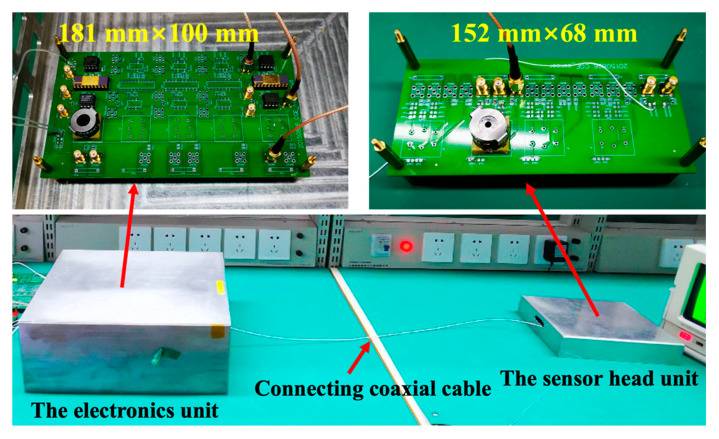
The prototype circuit of the capacitive position sensor in our laboratory.

The experiments are implemented on the prototype circuit in our laboratory. The experimental setup for the transmission cable investigation in the high-precision capacitive position sensor is shown in Figure 5. The whole system consists of a simulative sensor head unit and capacitive position detection circuits unit. The sensor head includes a transformer and the differential capacitance. The PCB size of this simulative sensor head unit is 152 mm (length) × 68 mm (width). The detection electronics includes the preamplifier, the band-pass filter, and the lock-in amplifier. The PCB size of the electronics unit is 181 mm (length) × 100 mm (width). The data acquisition unit adopts a 24-bit ADC device.

**Figure 6:**
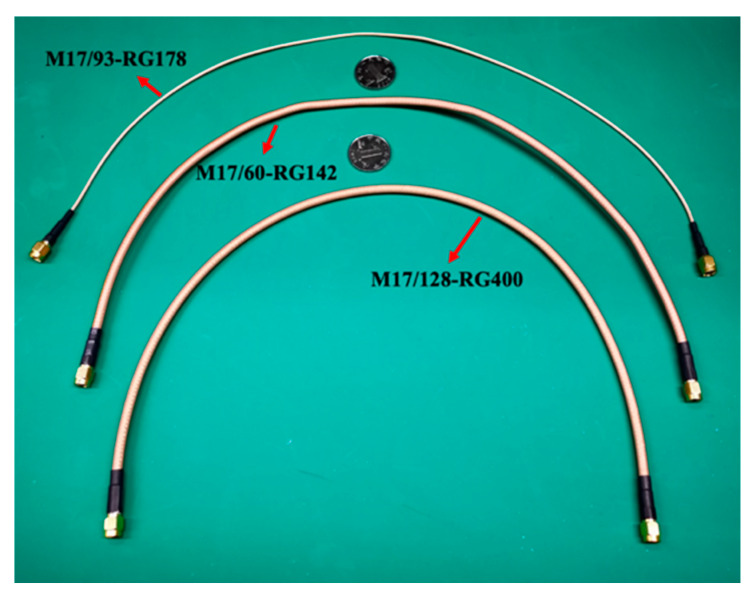
The different types of coaxial cables (0.5 m) used in our experiment.

In our experiments, three different types of coaxial cables produced by Thermax manufacturers are chosen in order to compare their performance. The types and the characters of coaxial cables are shown in Table 2. The material of the inner conductor is silver-coated copper. The material of the insulator between the inner and ground and between the ground to the external side is FEP (Fluorinated ethylene propylene). The nominal stray capacitance is about 29.4 pF/ft, namely 96.5 pF/m. The main difference is the conductor structure by using different stranding and diameter. Each type meanwhile is 0.5-m-long, 1-m-long, and 2-m-long, respectively. Figure 6 gives the photos of these cables.

### 3.2. Stray-Capacitance Estimation

The resonant capacitance of circuits is placed nearby the sensor head. It can make the output voltage noise of the front-end circuit achieves the minimum value at the center frequency through adjusting the resonant capacitance. Actually, a spectrum analyzer was used to measure the resonant frequency to assure the optimal resonant condition. According to the theoretical analysis, the frequency error of the spectrum analyzer and the actual experimental result of the lowest curve frequency, then the total equivalent resonance capacitance is 504 ± 6 pF.

According to Equation (5), the total resonance capacitance includes the tuning capacitance *C*_r_, which is a real capacitor soldered on the PCB, the residual stray-capacitance *C*_p_ of the PCB which could be assumed as stable because it is irrelevant to the cables and detection electronics, and the stray-capacitance of the cables.

Here, the total equivalent resonance capacitance, namely 2*C*_r_ + 2*C*_p_ + *k*_c_*l*_c_, is about 504 pF. The tuning capacitance *C*_r_ is actually measured shown in Table 3. We assume the value of the residual stray-capacitance *C*_p_ is constant and the stray-capacitance per unit length of these coaxial cables *k*_c_ is constant. Finally, by changing the types and three lengths of the cables, the stray-capacitance of the cables and the residual stray-capacitance of the PCB can be finally obtained by linear fitting method. Table 3 gives the stray-capacitance estimation results in our experiments. The uncertainty of the stray-capacitance comes from the fitting error, the testing error of the tuning capacitance, and the total equivalent resonance capacitance. It shows that the stray-capacitance of above three different cables is approximately the same, and the stray-capacitance per unit length of these coaxial cables *k*_c_ is 90.7 ± 1.3 pF/m, which is close to the nominal value.

**Figure 7:**
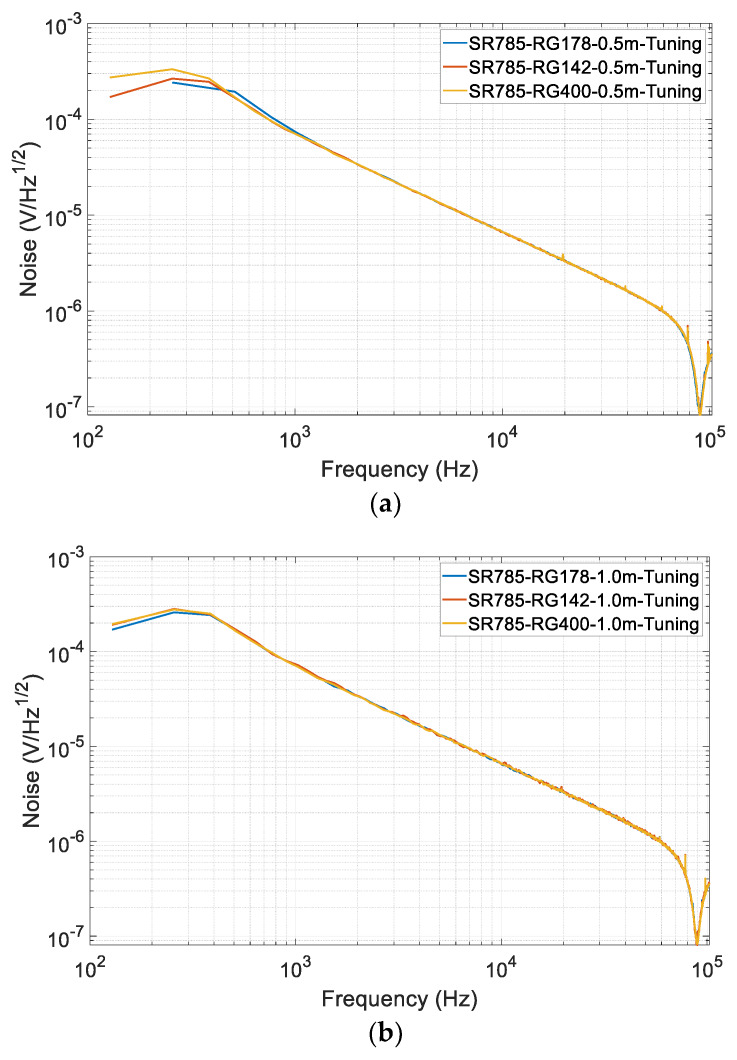

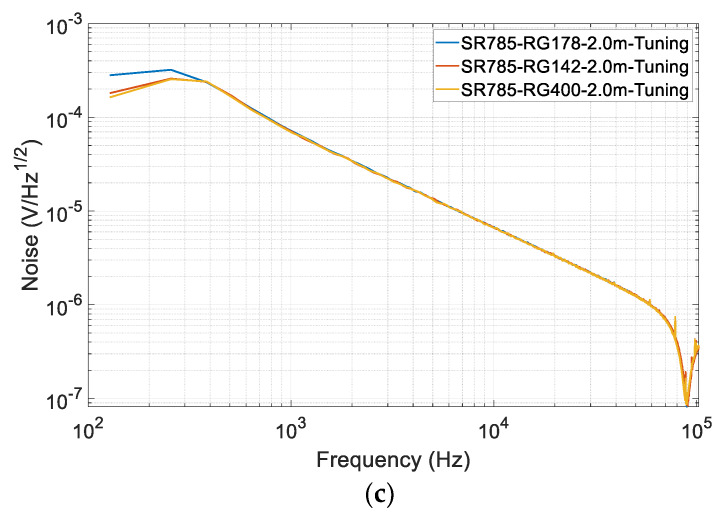
The measured output voltage noise curve of the preamplifier after the tuning under different lengths of the cables: (**a**) using the 0.5-m-long cable; (**b**) using the 1.0-m-long cable; (**c**) using the 2-m-long cable.

Each time after the tuning, a dynamic signal analyzer SR785 [31] which is manufactured by Stanford Research Systems was used to verify the resonant frequency and test the noise of the preamplifier. Figure 7 shows the measured output voltage noise curve of the preamplifier, where it achieves the minimum value of about 100 nV/Hz^1/2^ at 90 kHz under all circumstances, which agrees with the theoretical value. It also means the resonant condition is all met by additional tuning, then the emphasis is to compare their performance.

### 3.3. Calibration of the Whole Capacitive Transducer

**Figure 8:**
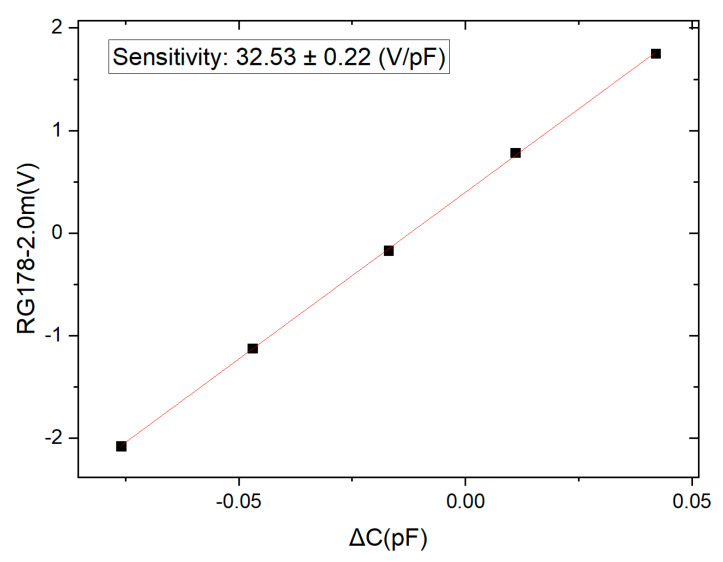
The calibration graph of using a 2-m-long RG178 type cable.

Before the noise level comparison of this circuit, first, an adjustable differential capacitance facility that is made in our lab was used to calibrate the whole capacitive transducer. Especially, Figure 8 gives the calibration graph by using the 2-m-long RG178 type cable. Table 4 shows the sensor’s linear fitting result of the sensitivity coefficient at all nine conditions. It indicates all the sensitivity is equal within the limits of experimental error. It also means in our connecting method, the attenuation of the signal by the cables could be neglected.

### 3.4. Noise Level Comparison of the Whole Capacitive Transducer

**Figure 9:**
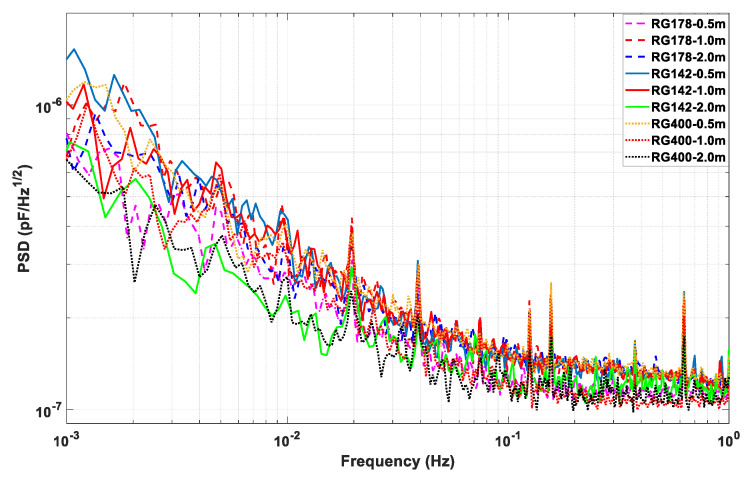
Noise level of the capacitive position sensor under all nine circumstances.

Finally, the performance of the circuit which works under all the above circumstances is given. First, the front-end circuit is tuned and the whole capacitive sensor is calibrated. Second, the ADC device is used to acquire the voltage data of the sensor, and the noise power spectrum density of the voltage is analyzed. Finally, the voltage noise divides each corresponding calibrated sensitivity coefficient presented in Table 4, in which the capacitance detection noise level with the unit of pF/Hz^1/2^ can be obtained. The experimental results are compared in Figure 9, which shows that the capacitance detection noise level does not have obvious differences between different cables.

## 4. Discussions

From the above analysis and experimental investigation of the stray-capacitance influences of the cables, Figure 9 shows that all the noise level is about 1–2 × 10^−7^ pF/Hz^1/2^ at 0.1 Hz and 3–5 × 10^−7^ pF/Hz^1/2^ at 6 mHz which also meets the requirements of the satellite Earth’s gravity field recovery missions and TianQin space gravitational wave detection mission [5].

In order to reduce the influences and achieve low-noise capacitive position measurement, basic design factors are given here. First, the resonant condition equation should be met experimentally to obtain the best noise level. It means the resonant frequency and the length of the cable could be flexibly adjusted by changing the tuning capacitance. Second, the whole circuit needs to be calibrated and tested after choosing the transmission cable and the tuning. The smaller of the stray capacitance per unit length of the cable, the better to increase the transmission length. However, there exists an upper limit of the length at the pumping frequency.

Though this work verified the feasibility of the connecting method, the other influences of the cables, such as the long-term stability and the temperature effect, need to be further carefully studied in the future.

## 5. Conclusions

In this work, a low-noise capacitive position sensor with a coaxial cable connecting the sensor head and the electronics has been designed and realized for its space applications in satellite Earth’s gravity field recovery missions and space gravitational wave detection missions. The stray-capacitance influences of the cables are studied experimentally. The whole capacitive transducer is tuned and calibrated by using three different types and lengths of coaxial cables. The experimental results show that the influence of the cables’ stray-capacitance could be ignored for engineering consideration under the resonant condition and it indicates that using a coaxial cable between the preamplifier and the three-winding transformer that is placed near the core is an effective way to detect the TM motion for engineering applications.

## Figures and Tables

**Table 1:** Main typical parameters of the capacitive transducer.

Electronic Parameters	Value
Transformer inductance *L*	6.3 mH
Transformer quality factor *Q*_t_	~90
Equivalent coaxial quality factor *Q*_c_	~3.4 × 10^4^
Feedback capacitance *C*_f_	5.0 pF
Input noise voltage density *e*_n_	15.0 nV/Hz^1/2^
Input noise current density *i*_n_	10.0 fA/Hz^1/2^
AC pumping amplitude *V*_p_	5.0 V
AC pumping frequency *ω*_p_	2π × 90 kHz
Coaxial cables	In Table 2

**Table 2:** The types and main characters of three coaxial cables.

Types	Conductor Stranding	Conductor Diameter	Cable Outer Diameter	Stray Capacitance
M17/60-RG142	1	0.91 mm	4.95 mm	96.5 pF/m
M17/93-RG178	7	0.30 mm	1.80 mm	96.5 pF/m
M17/128-RG400	19	0.98 mm	4.95 mm	96.5 pF/m

**Table 3:** The stray-capacitance estimation under different length of the cables.

Cables	Length	The Tuning Capacitance *C*_r_	PCB Stray-Capacitance *C*_p_	Cable’s Stray-Capacitance *C*_c_
RG142/RG178/RG400	0.5 m	204.2 ± 0.5 pF	24.9 ± 0.8 pF	45.4 ± 0.7 pF
	1.0 m	182.4 ± 0.5 pF		90.7 ± 1.3 pF
	2.0 m	136.3 ± 0.5 pF		181.4 ± 2.6 pF

**Table 4:** The sensors’ calibrated sensitivity under nine conditions.

Cables	Calibrated Sensitivity Coefficient (V/pF)		
	0.5 m	1.0 m	2.0 m
RG142	31.37 ± 0.26	32.33 ± 0.21	32.16 ± 0.21
RG178	31.53 ± 0.21	32.46 ± 0.21	32.53 ± 0.22
RG400	31.68 ± 0.20	32.52 ± 0.21	32.49 ± 0.21

## References

[1] Touboul P., Foulon B., Christophe B., Marque J.P., Steve Kenyon S., Pacino M.C., Marti U. (2012). CHAMP, GRACE, GOCE instruments and beyond. Geodesy for Planet Earth.

[2] Armano M., Audley H., Baird J., Binetruy P., Born M., Bortoluzzi D., Castelli E., Cavalleri A., Cesarini A., Cruise A.M. (2018). Beyond the Required LISA Free-Fall Performance: New LISA Pathfinder Results down to 20 μHz. Phys. Rev. Lett..

[3] Armano M., Audley H., Auger G., Baird J.T., Bassan M., Binetruy P., Born M., Bortoluzzi D., Brandt N., Caleno M. (2016). Sub-Femto-g Free Fall for Space-Based Gravitational Wave Observatories: LISA Pathfinder Results. Phys. Rev. Lett..

[4] Touboul P., Métris G., Rodrigues M., André Y., Baghi Q., Bergé J., Boulanger D., Bremer S., Carle P., Chhun R. (2017). MICROSCOPE Mission: First Results of a Space Test of the Equivalence Principle. Phys. Rev. Lett..

[5] Luo J., Chen L., Duan H., Gong Y., Hu S., Ji J., Liu Q., Mei J., Milyukov V., Sazhin M. (2016). TianQin: A Space-Borne Gravitational Wave Detector. Classical Quant. Grav..

[6] Josselin V., Touboul P., Kielbasa R. (1999). Capacitive Detection Scheme for Space Accelerometers Applications. Sens. Actuators A Phys..

[7] Mance D. (2012). Development of Electronic System for Sensing and Actuation of Test Mass of the Inertial Sensor LISA. Ph.D. Thesis.

[8] Cavalleri A., Dolesi R., Fontana G., Hueller M., Turneaure J., Vitale S., Weber W. (2001). Progress in the Development of a Position Sensor for LISA Drag-Free Control. Classical Quant. Grav..

[9] Gan L., Mance D., Zweifel P. (2011). Actuation to Sensing Crosstalk Investigation in the Inertial Sensor Front-End Electronics of the Laser Interferometer Space Antenna Pathfinder Satellite. Sens. Actuators A Phys..

[10] Gan L., Mance D., Zweifel P. (2012). LTP is FEE Sensing Channel: Front-End Modeling and Symmetry Adjustment Method. IEEE Sens. J..

[11] Armano M., Audley H., Auger G., Baird J., Bassan M., Binetruy P., Born M., Bortoluzzi D., Brandt N., Caleno M. (2017). Capacitive Sensing of Test Mass Motion with Nanometer Precision Over Millimeter-Wide Sensing Gaps for Space-Borne Gravitational Reference Sensors. Phys. Rev. D.

[12] Armano M., Audley H., Baird J., Born M., Bortoluzzi D., Cardines N., Castelli E., Cavalleri A., Cesarini A., Cruise A.M. (2020). Analysis of the Accuracy of Actuation Electronics in the Laser Interferometer Space Antenna Pathfinder. Rev. Sci. Instrum..

[13] Bai Y., Zhou Z., Tu H., Wu S., Cai L., Liu L., Luo J. (2009). Capacitive Position Measurement for High-Precision Space Inertial Sensor. Frontiers of Physics in China.

[14] Hu M., Bai Y.Z., Zhou Z.B., Li Z.X., Luo J. (2014). Resonant Frequency Detection and Adjustment Method for a Capacitive Transducer with Differential Transformer Bridge. Rev. Sci. Instrum..

[15] Xie Y., Fan J., Zhao C., Yan S., Hu C., Tu L. (2019). Modeling and Analysis of the Noise Performance of the Capacitive Sensing Circuit with a Differential Transformer. Micromachines.

[16] Li Z., Zhang X., Zou S., Huang X., Xue C., Liu J., Liu Q., Yang S., Tu L. (2020). Design of a Carrier Wave for Capacitive Transducer with Large Dynamic Range. Sensors.

[17] Siemes C., Rexer M., Schlicht A., Haagmans R. (2019). GOCE Gradiometer Data Calibration. J. Geod..

[18] Floberghagen R., Fehringer M., Lamarre D., Muzi D., Frommknecht B., Steiger C., Piñeiro J., Da Costa A. (2011). Mission Design, Operation and Exploitation of the Gravity Field and Steady-State Ocean Circulation Explorer Mission. J. Geod..

[19] Lotters J.C., Olthuis W., Veltink P.H., Bergveld P. (1999). A Sensitive Differential Capacitance to Voltage Converter for Sensor Applications. IEEE T. Instrum. Meas..

[20] Zhao Y., Zhao J., Wang X., Xia G.M., Qiu A.P., Su Y., Xu Y.P. (2015). A Sub-Μg Bias-Instability MEMS Oscillating Accelerometer with an Ultra-Low-Noise Read-Out Circuit in CMOS. IEEE J. Solid-St. Circ..

[21] Gilavdary I., Mekid S., Riznookaya N., Abdul Sater A. (2018). Transient Responses and Stability in the Differential Electrostatic Sensor of Inertial and Gravitational Moments with Asymmetry. Measurement.

[22] Verma V.K., Yadava R.D.S. (2016). Stochastic Resonance in MEMS Capacitive Sensors. Sens. Actuators B Chem..

[23] Yin Y., Sun B., Han F. (2016). Self-Locking Avoidance and Stiffness Compensation of a Three-Axis Micromachined Electrostatically Suspended Accelerometer. Sensors.

[24] Gettings C., Speake C.C. (2019). A Method for Reducing the Adverse Effects of Stray-Capacitance on Capacitive Sensor Circuits. Rev. Sci. Instrum..

[25] Sunderland A., Veryaskin A.V., Golden H., Mcrae W., Ju L., Blair D.G. (2009). Differential Readout for a Magnetic Gradiometer. Sens. Actuators A Phys..

[26] Baikie I.D., Venderbosch E., Meyer J.A., Estrup P.J.Z. (1991). Analysis of Stray Capacitance in the Kelvin Method. Rev. Sci. Instrum..

[27] Nagatomo T., Miki N. (2018). Reduction of Parasitic Capacitance of a PDMS Capacitive Force Sensor. Micromachines.

[28] Kim H., Lee B., Mun Y., Kim J., Han K., Roh Y., Song D., Huh S., Ko H. (2018). Reconfigurable Sensor Analog Front-End Using Low-Noise Chopper-Stabilized Delta-Sigma Capacitance-to-Digital Converter. Micromachines.

[29] Xu H., Qiao J., Zhang J., Han H., Li J., Liu L., Wang B. (2018). A High-Resolution Leaky Coaxial Cable Sensor Using a Wideband Chaotic Signal. Sensors.

[30] De Marcellis A., Reig C., Cubells-Beltrán M. (2019). A Capacitance-to-Time Converter-Based Electronic Interface for Differential Capacitive Sensors. Electronics.

[31] SRS Data Sheets. https://thinksrs.com/downloads/pdfs/catalog/SR785c.pdf.

